# GenomeWarp: an alignment-based variant coordinate transformation

**DOI:** 10.1093/bioinformatics/btz218

**Published:** 2019-03-27

**Authors:** Cory Y McLean, Yeongwoo Hwang, Ryan Poplin, Mark A DePristo

**Affiliations:** 1 Verily Life Sciences, Mountain View, CA, USA; 2 Google Inc., Mountain View, CA, USA; 3 Department of Computer Science, Carnegie Mellon University, Pittsburgh, PA, USA

## Abstract

**Summary:**

Reference genomes are refined to reflect error corrections and other improvements. While this process improves novel data generation and analysis, incorporating data analyzed on an older reference genome assembly requires transforming the coordinates and representations of the data to the new assembly. Multiple tools exist to perform this transformation for coordinate-only data types, but none supports accurate transformation of genome-wide short variation. Here we present GenomeWarp, a tool for efficiently transforming variants between genome assemblies. GenomeWarp transforms regions and short variants in a conservative manner to minimize false positive and negative variants in the target genome, and converts over 99% of regions and short variants from a representative human genome.

**Availability and implementation:**

GenomeWarp is written in Java. All source code and the user manual are freely available at https://github.com/verilylifesciences/genomewarp.

**Supplementary information:**

Supplementary data are available at *Bioinformatics* online.

## 1 Introduction

The Human Genome Project produced the first full draft of the human genome sequence (International Human Genome Sequencing Consortium, 2001). Since then, the assembly of the human genome has been refined and updated multiple times (International Human Genome Sequencing Consortium, 2004). Higher quality reference genome sequences improve the mapping and alignment of sequence read data, but present challenges for integrating data mapped to other genome assembly versions.

The task of converting genomic regions between genome assemblies, known as *lift over*, is performed by creating gapped pairwise alignment *chains* (Kent *et al.*, 2003) between the assemblies and then transforming the region coordinates based on those chains. Many tools perform genomic region lift over, including UCSC LiftOver (Kuhn *et al.*, 2013) and CrossMap (Zhao *et al.*, 2014). These tools support lift over of multiple data formats, with CrossMap supporting Binary Alignment Map, Browser Extensible Data, BigWig, General Feature Format, Gene transfer format, Sequence Alignment Map, Wiggle and Variant Call Format (VCF).

An unsupported data type of particular interest is genome-wide variation, in which both variations with respect to the reference assembly and regions that confidently match the reference assembly are encoded. These data are semantically distinct from VCF, as they allow disambiguation between regions in which genotypes are unknown and those that confidently match the reference. As such, genome-wide variation data attempt to represent an individual’s entire genome sequence, encoded with respect to the reference. Genome-wide variation data are often formatted as a Genome VCF (gVCF) file, which encodes variant sites and confidently called regions of the genome in distinct rows. Many popular variant callers, including DeepVariant (Poplin *et al.*, 2018) and GATK HaplotypeCaller (Van der Auwera *et al.*, 2013), emit gVCF output and gVCF files are widely used as input to joint genotyping algorithms (Lin *et al.*, 2018; Poplin *et al.*, 2017).

**Fig. 1.**
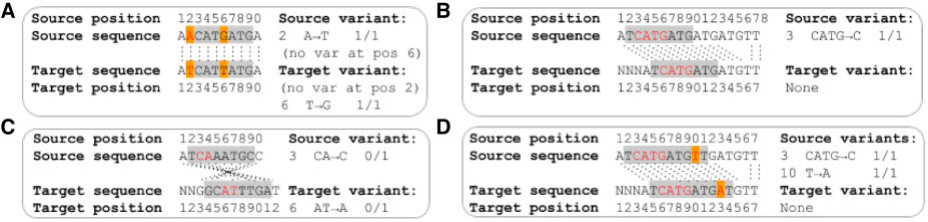
Algorithmic issues encountered when mapping variants between assemblies. Gray boxes indicate confidently called regions. Orange boxes indicate reference genome differences between assemblies. Red letters indicate reported variants in the source genome and their corresponding base pairs in the target genome. Homologous base pairs in the source and target genomes are joined by dotted black lines. (**A**) Reference sequence changes across genome assemblies can create or remove variants. (**B**) Indel variant representations can be affected by sequence outside the confidently called regions. The homozygous loss of ‘ATG’ in the source genome matches the removal of that sequence in the target genome. (**C**) Opposite strand alignments can cause indel representation changes. Since indels are left-aligned by convention, when strands are flipped the reference anchor base moves to the other side of the indel. This may also cause the indel location to change. (**D**) Indel and single nucleotide polymorphism variants can interact with each other within a single confident region

Translating genome-wide variation data between genome assemblies is more complex than coordinate-only transformations owing to changes in the sequence content between genome assemblies (Fig. 1). Here we describe GenomeWarp, a tool for converting genome-wide short variation data between genome assemblies. Its algorithm is tuned to minimize false positive and negative variants induced by transformation, by marking regions that cannot be guaranteed to transform correctly as unknown. When realigning and recalling variants in a target genome is infeasible, GenomeWarp can accurately convert callsets across genome assemblies.

## 2 Materials and methods

The workflow of GenomeWarp is as follows (Supplementary Fig. S1): an input gVCF is modified into source variants and confidently called source regions. The regions are preprocessed to contain only canonical DNA characters by splitting any regions that contain ambiguous bases into non-overlapping regions that exclude those characters. The resulting source regions are then lifted over to the target assembly via a chain file of pairwise alignments, resulting in raw target region outputs. Because chain files can map multiple regions in the source assembly to a single region in the target assembly, target regions are post-processed to omit overlapping regions (Supplementary Fig. S2). For each confidently called region that is lifted over to the target assembly, all variant records within the region are collectively considered jointly with the reference sequences to transform the representations into the set of target assembly variants that reflect the same sequence content.

Many edge cases must be handled to accurately transform variants within a confidently called region from a source assembly to a target assembly (Fig. 1). The general transformation algorithm requires creating individual haplotypes based on the source and resolving them with respect to the target (Supplementary Fig. S3). However, because the human genome assemblies are quite similar in mapped sequence content (Supplementary Table S1), the general algorithm is rarely needed in practice and simpler transformations can be applied in common cases. GenomeWarp classifies regions based on reference genome composition, whether the homologous regions between assemblies are on the same genome strand, and whether the region contains any insertion/deletion (indel) variants (Supplementary Table S2). A subset of all region type transformations is supported in GenomeWarp v1.2; regions that require haplotype alignment are not transformed. By avoiding alignment, the algorithm does not have to match the alignment parameters used in the original chain file. Unsupported transformations cause the associated confidently called region and its constituent variants to be omitted, effectively turning them into unknown regions. This ensures that the final output of GenomeWarp accurately reflects all variants within the confidently called regions present in the target assembly.

The utility of GenomeWarp is demonstrated by its conversion of HG001, the pilot benchmark callset of the Genome in a Bottle Consortium (GiaB) (Zook *et al.*, 2014), from the GRCh37 to the GRCh38 assembly (Supplementary Table S3). While the GiaB benchmarking regions are likely easier to transform than regions of higher complexity, this should affect performance of all transformation tools. Over 99.9% of benchmarking regions whose coordinates can be lifted over to GRCh38 are successfully transformed, along with 99.4% of single nucleotide variants and 98.7% of indels. Compared to existing conversion methods, GenomeWarp reduces erroneous single nucleotide polymorphisms 19–35-fold and erroneous indels 9–10-fold (Supplementary Note). Indeed, GenomeWarp was used in the generation of subsequent GiaB GRCh38 reference materials for Complete Genomics, Ion Torrent and SOLiD data (Zook *et al.*, 2019). GenomeWarp completed the conversion using one 2.8 GHz core and 20 GB RAM in 13 min, in contrast to the hundreds of core hours required to align reads and call variants directly. Memory and compute resources scale linearly in the number of regions and variants in the source assembly, and work can be sharded across chromosomes to reduce the total RAM required.

The gold standard methodology for identifying variation in a genome assembly is to align reads to that assembly and call variants based on those reads. However, this gold standard may not be possible if the raw reads no longer exist or are otherwise unavailable for analysis. Realigning and recalling variants may also be impractical for computational or cost considerations. In these cases, GenomeWarp provides a computationally efficient mechanism to accurately transform genome-wide short variation data from one assembly to another.

## Supplementary Material

btz218_Supplementary_Material
