# Supplementary material for: GenomeWarp: an alignment-based variant coordinate transformation
Source: Bioinformatics. 2019 Mar 27;35(21):4389–91. doi: 10.1093/bioinformatics/btz218 (PMC6821237; doi:10.1093/bioinformatics/btz218)
Supplement: btz218_Supplementary_Material [file btz218_supplementary_material.pdf]

Supplementary Material for GenomeWarp: an alignment-based variant coordinate transformation

Cory Y. McLean[1,2,*], Yeongwoo Hwang[1,3,†], Ryan Poplin[1,2], Mark DePristo[1,2]

[1] Verily Life Sciences, 1600 Amphitheatre Pkwy, Mountain View, CA 94043, USA
[2] Google Inc., 1600 Amphitheatre Pkwy, Mountain View, CA 94043, USA
[3] Carnegie Mellon University, 5000 Forbes Ave, Pittsburgh, PA 15213, USA
[†] Work performed while an intern at Verily Life Sciences.
[*] To whom correspondence should be addressed: cym@google.com

Supplementary Figures 2

Supplementary Tables 5

Supplementary Note 13

Supplementary References 18

Supplementary Figures

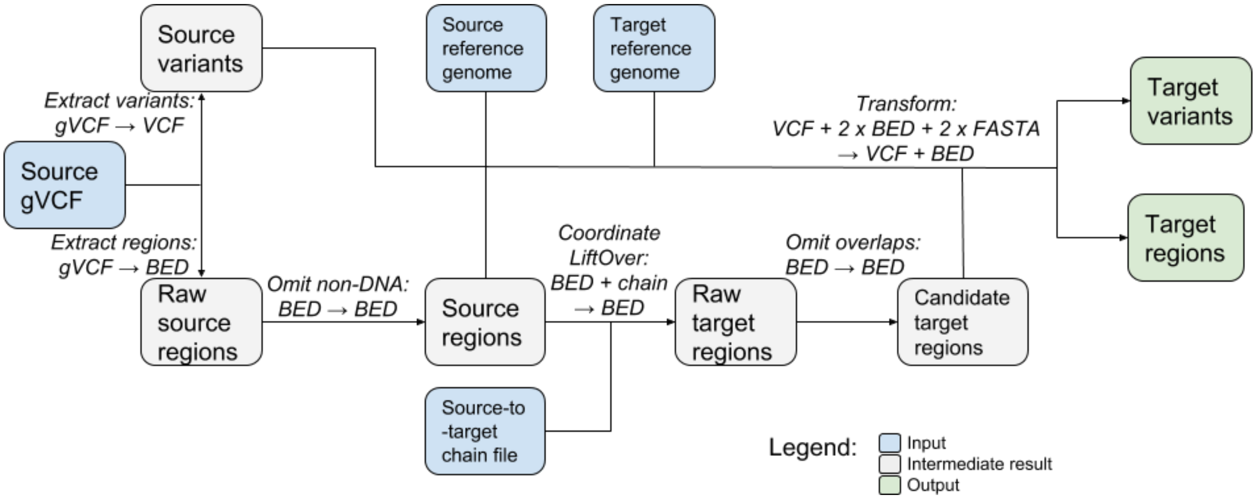

Supplementary Figure S1: **Overview of the GenomeWarp algorithm.** Transformations applied between data are labeled arrows. Chain files generated by UCSC are available for download for each query genome at http://hgdownload.cse.ucsc.edu/goldenPath/<query_genome>/liftOver/.

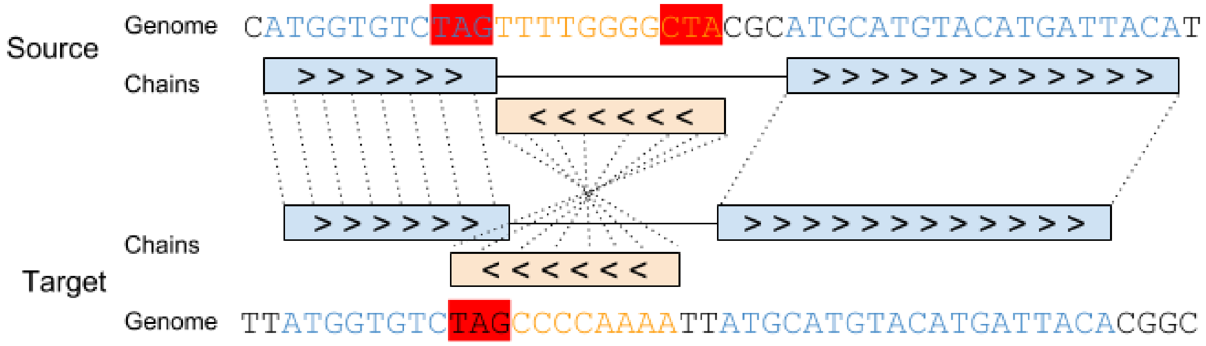

Supplementary Figure S2: **Non-overlapping source assembly regions can produce overlapping target assembly regions.** Matching sequences on the positive strand are shown in blue. Matching sequences on the negative strand are shown in orange. A single target assembly region that is mapped from multiple source regions is highlighted in red in both the source and target assemblies: base pairs 2-12 in the source genome are identical to base pairs 3-13 in the target genome on the same strand, while base pairs 13-23 in the source genome are identical to base pairs 11-21 in the target genome on the opposite strand. Since the three target genome base pairs 11-13 are linked to multiple distinct source genome regions, the regions are omitted from further consideration by GenomeWarp. The chain diagram uses the UCSC Genome Browser Chain representation.

For each confidently called region in the source genome:

1. Identify all variants within the region.
2. Create haplotypes based on both the region sequence in the source genome and the variants within the region.
3. Align the haplotypes to the region sequence in the target genome.
4. Emit variants in the target genome based on the haplotype alignments.
5. Emit the target genome region coordinates as a confidently called region.

Supplementary Figure S3: Pseudocode for the general algorithm to perform variant conversion between assemblies. A GenomeWarp implementation based on the above pseudocode could handle all possible interactions between variants and reference genome differences. However, because human genome assemblies are quite similar in mapped sequence content (**Supplementary Table S1**), GenomeWarp version 1.2 relies on a set of simpler heuristics (**Supplementary Table S2**) that account for nearly all regions seen in practice.

Supplementary Tables

Supplementary Table S1: Distribution of HG001 GiaB v3.3.2 GRCh37 source SNPs and biallelic indels classified by region type when transforming to GRCh38. Of the 3,775,119 total variants in the GiaB HG001 benchmarking callset on GRCh37, 3,258,078 are SNPs and 478,217 are biallelic indels. These are classified into different region types based on the base pair composition of the homologous regions of the source and target genome assemblies. "Identical" indicates the same number and order of nucleotides; "Mismatch" indicates the same number of nucleotides in the source and target genomes but at least one mismatch between the sequences; "Alignment" indicates the number of nucleotides in the source and target genomes differ. "Unmapped" indicates source variants either not wholly within benchmarking source regions or that could not be uniquely mapped to the target genome assembly with all bases remapping. "+" and "-" indicate that the region maps to GRCh38 on the forward and reverse strand, respectively.

Variant type	Region Type	Strand	Number of variants
SNP	Identical	+	3,187,530
		-	2,062
	Mismatch	+	16,135
		-	475
	Alignment	+	43
		-	2
	Unmapped	N/A	51,831
Biallelic indel	Identical	+	443,630
		-	195
	Mismatch	+	27
		-	0
	Alignment	+	1,897
		-	31
	Unmapped	N/A	32,437

Supplementary Table S2: **Summary of variant types and the method needed to resolve changes.** Region type and strand definitions are defined in **Supplementary Table S1** above.

**Region Type**	**Strand**	**Has indel?**	**Realignment required?**	**Supported in GenomeWarp v1.2?**	**Notes**
Identical	+	No	No	Yes	The most straightforward case: a simple coordinate transformation. All variants within a region retain the same reference and alternate alleles, and all genotypes remain the same.
		Yes	No	Yes	In most cases, this is also a simple coordinate transformation, where all variants within a region retain the same reference and alternate alleles. However, some cases may be influenced by a reference genome change outside the region (see Figure 1B for an example where the reference genome change absorbs a homozygous reference variant).
	-	No	No	Yes	Straightforward coordinate transformation and allele complementation. All variants within a region have each allele changed to its reverse complement sequence, and all genotypes remain the same.
		Yes	No	No	Requires left alignment and anchor allele change. May be influenced by reference genome change outside the region (see Figure 1C).
Mismatch	+	No	No	Yes	Coordinate transformation,

					and may need to change allele counts if it coincides with a reference genome change (Figure 1A). All positions that differ between the reference genomes are modified. If no variant is present in the source genome (i.e. homozygous reference) the resulting variant is homozygous alternate in the target genome. If a homozygous variant allele in the source genome matches the target genome reference sequence it is no longer represented (Figure 1A). In other cases, the reference allele is changed to reflect the target genome base pair(s).
		Yes	Yes	No	Indel and reference sequence changes can interact in complex ways to affect variant representations. See Figure 1D.
	-	No	No	Yes	Coordinate transformation and allele complementation. May need to change allele counts if it coincides with a reference genome change.
		Yes	Yes	No	Requires left alignment and anchor allele change. Similar to Figure 1D, indel and reference sequence changes can interact in complex ways to affect variant representations.
Alignment*	+	No	Yes	No	Since LiftOver chains do not provide alignments,

					source and target sequence must be aligned.
		Yes	Yes	No	Since LiftOver chains do not provide alignments, source and target sequence must be aligned.
	-	No	Yes	No	Since LiftOver chains do not provide alignments, source and target sequence must be aligned.
		Yes	Yes	No	Since LiftOver chains do not provide alignments, source and target sequence must be aligned.

* GenomeWarp version 1.2 does not support transformation of any regions that require alignment; while in principle this would provide a single method for transforming all regions, in practice the additional algorithmic and computational complexity are not warranted for the small number of regions this affects (**Supplementary Table S1**).

Supplementary Table S3: hap.py evaluation of GenomeWarp, CrossMap, and Picard LiftoverVcf conversion of HG001 GiaB v3.3.2 from GRCh37 to GRCh38. The best performing tool in each column is highlighted in bold. NCBI Remap did not successfully convert the variants so is excluded from the comparisons.

Algorithm	Total	TP	FN	FP	Recall	Precision	F1
**SNP performance**							
GenomeWarp	2971767	**2971675**	**92**	292	**0.999969**	0.999902	**0.999935**
CrossMap	2976034	2964008	12026	1487	0.995959	0.999499	0.997726
Picard LiftoverVcf	2976034	2965268	10766	**219**	0.996382	**0.999926**	0.998151
Picard LiftoverVcf `RECOVER_SWAPPED_REF_ALT=true`	2976034	2968911	7123	295	0.997607	0.999901	0.998752
**Indel performance**							
GenomeWarp	439693	439360	**333**	**32**	**0.999243**	**0.999930**	**0.999586**
CrossMap	441354	439642	1712	1941	0.996121	0.995803	0.995962
Picard LiftoverVcf	441354	439679	1675	1774	0.996205	0.996163	0.996184
Picard LiftoverVcf `RECOVER_SWAPPED_REF_ALT=true`	441354	**439680**	1674	1773	0.996207	0.996165	0.996186

Using the terminology that *true variants* are those in the HG001 GiaB v3.3.2 benchmarking callset on GRCh38, and *called variants* are those present in the callset produced by taking the HG001 GiaB v3.3.2 benchmarking callset on GRCh37 and transforming it to GRCh38 using the tool of interest, the above table uses the following definitions:
TP: true positive variants, i.e. variants that are in both the *true variants* and *called variants* callsets;
FN: false negative variants, i.e. variants that are in the *true variants* callset but not the *called variants* callset;
FP: false positive variants, i.e. variants that are not in the *true variants* callset but are in the *called variants* callset.

Supplementary Table S4: Data used to convert HG001 GiaB v3.3.2 from GRCh37 to GRCh38.

File description	URL
GRCh37 (source) BED of benchmarking regions	https://ftp-trace.ncbi.nlm.nih.gov/giab/ftp/release/NA12878_HG001/NISTv3.3.2/GRCh37/HG001_GRCh37_GIAB_highconf_CG-IllFB-IllGATKHC-Ion-10X-SOLID_CHROM1-X_v.3.3.2_highconf_nosomaticdel.bed
GRCh37 (source) VCF	https://ftp-trace.ncbi.nlm.nih.gov/giab/ftp/release/NA12878_HG001/NISTv3.3.2/GRCh37/HG001_GRCh37_GIAB_highconf_CG-IllFB-IllGATKHC-Ion-10X-SOLID_CHROM1-X_v.3.3.2_highconf_PGandRTGphasetransfer.vcf.gz
GRCh37 (source) genome assembly	http://ftp.1000genomes.ebi.ac.uk/vol1/ftp/technical/reference/human_g1k_v37.fasta.gz
GRCh38 (target) genome assembly	ftp://ftp.1000genomes.ebi.ac.uk/vol1/ftp/technical/reference/GRCh38_reference_genome/GRCh38_full_analysis_set_plus_decoy_hla.fa
LiftOver chain file	http://hgdownload.cse.ucsc.edu/goldenPath/hg19/liftOver/hg19ToHg38.over.chain.gz

Supplementary Table S5: hap.py evaluation of GiaB HG001 benchmarking callset on GRCh38 transformed to hs37d5 and back to GRCh38 using GenomeWarp, evaluated against the GiaB HG001 benchmarking callset on GRCh38. Columns are defined as described in **Supplementary Table S3**. All false positives are due to the non-injective nature of liftover chains (see **Supplementary Note** for details).

**Variant type**	**Total**	**TP**	**FN**	**FP**	**Recall**	**Precision**	**F1**
SNP	3042783	3032901	9882	43	0.996752	0.999986	0.998366
Indel	499697	496242	3455	1	0.993086	0.999998	0.99653

Supplementary Table S6: hap.py evaluations of HG003 mapped to both hs37d5 and GRCh38. Columns are defined as described in **Supplementary Table S3**.

**Variant type**	**Total**	**TP**	**FN**	**FP**	**Recall**	**Precision**	**F1**
Variants called on GRCh38, evaluated against GiaB benchmark callset on GRCh38							
SNP	2882677	2881797	880	2259	0.999695	0.999217	0.999456
Indel	471482	465624	5858	2521	0.987575	0.994815	0.991182
Variants called on hs37d5, evaluated against GiaB benchmark callset on hs37d5							
SNP	3012250	3011588	662	1234	0.99978	0.999591	0.999685
Indel	459708	456665	3043	1726	0.993381	0.996378	0.994877
Variants called on hs37d5, GenomeWarped to GRCh38, evaluated against GiaB benchmark callset on GRCh38							
SNP	2882677	2870490	12187	3222	0.995772	0.998879	0.997323
Indel	471482	462981	8501	2600	0.98197	0.994623	0.988256
Variants called on hs37d5, GenomeWarped to GRCh38, evaluated against variants called with DeepVariant on GRCh38 directly in areas with non-variant site GQ >= 20							
SNP	3971123	3755862	215261	27802	0.945793	0.992663	0.968662
Indel	879297	842238	37059	11199	0.957854	0.987414	0.972409

Supplementary Note

Comparison of GenomeWarp to other tools for transforming HG001

The National Institute of Standards and Technology hosts the Genome in a Bottle (GiaB) Consortium, which aims to provide reference genome materials for benchmarking purposes (Zook et al., 2014; Zook et al., 2016, Krusche et al, 2019). To evaluate the performance of GenomeWarp, the pilot genome HG001, benchmarking callset version v3.3.2 was transformed from the GRCh37 genome assembly to GRCh38 (all source data available from the links in **Supplementary Table S4**). Input files were modified to use consistent contig naming (e.g. “chr1” rather than “1”), then GenomeWarp v1.1.0 was run with its default parameters.

The benchmarking regions in the GRCh37 assembly span 2,575,064,465 base pairs (bp) and the GRCh37 VCF contains 3,775,119 variants, of which 3,690,061 are contained within a benchmarking region. Upon transformation to GRCh38, the benchmarking regions span 2,570,662,227 bp (99.8% of the source assembly) and the GRCh38 VCF contains 3,676,373 variants (99.6% as many variants as contained within benchmarking regions in the source assembly). The subset of region type transformations supported in GenomeWarp v1.1.0 are sufficient to capture this large number of input variants owing to the large fraction of regions containing identical base pairs on the same strand in the human genome assemblies (**Supplementary Table S1**).

The GiaB consortium directly created an HG001 v3.3.2 version of variants and benchmarking regions on GRCh38 as well as GRCh37. These GRCh38 data were used as a ground truth set to evaluate variants generated by lifting over the GRCh37 variants and regions using GenomeWarp and other lift over alternatives. It is important to note that differences between the lifted over GRCh37 variants and the native GRCh38 variants may arise due to multiple factors: 1) errors in lifting over variants between assemblies, and 2) callset differences based on the mapping and variant calling workflows performed on each assembly. While the latter source of errors is unavoidable, we expect their contributions to be relatively small owing to the similar sequence content of GRCh37 and GRCh38.

The alternative lift over tools analyzed were CrossMap (Zhao et al., 2014), Picard LiftoverVcf v2.18.14 (http://broadinstitute.github.io/picard/), and NCBI Remap. Since each of these tools operates solely on VCF files, the benchmarking regions were converted to GRCh38 using UCSC LiftOver (http://genome.ucsc.edu/cgi-bin/hgLiftOver). CrossMap was called with its default parameters. Picard LiftoverVcf was called both with default parameters and with the inclusion of the `RECOVER_SWAPPED_REF_ALT=true` flag. Multiple attempts to use NCBI Remap

(http://www.ncbi.nlm.nih.gov/genome/tools/remap) to map the GRCh37 VCF to GRCh38 all failed after VCF upload with an *HTTP 403 Forbidden* error.

For evaluation purposes, the truth regions were defined as the intersection of the GiaB v3.3.2 GRCh38 benchmarking regions and the regions lifted over from GRCh37 by the evaluated algorithm. The GiaB v3.3.2 GRCh38 benchmarking regions span 2,439,052,101 bp. The truth regions generated for GenomeWarp span 2,405,829,856 bp (98.6%). The truth regions generated using UCSC LiftOver span 2,408,207,454 bp (98.7%), slightly more than GenomeWarp owing to the conservative nature of GenomeWarp's conversion algorithm.

Variant accuracy was quantified using hap.py (https://github.com/illumina/hap.py), a haplotype VCF comparison tool, using the `vcfeval` engine. For SNPs, GenomeWarp identifies 2,764-7,667 more true positive variants than other tools and reduces false negatives 77- to 130-fold (**Supplementary Table S3**). For indels, GenomeWarp reduces false negatives 5-fold and reduces false positives 55- to 60-fold (**Supplementary Table S3**). The increase in, and similar total number of, false positive SNP calls for both GenomeWarp and Picard LiftoverVcf `RECOVER_SWAPPED_REF_ALT=true` compared to Picard LiftoverVcf without handling reference sequence changes indicates that those false calls are likely callset differences between GRCh37 and GRCh38 rather than logical errors introduced by the lift over algorithms, as the code to transform reference and alternate bases is only supported for biallelic SNPs by Picard and is very straightforward (see https://github.com/broadinstitute/picard/blob/master/src/main/java/picard/vcf/LiftoverVcf.java#L502-L514).

Differentiating between reference callset differences and GenomeWarp transformation errors

As illustrated in the above section, false positive and negative variants that arise when comparing a callset initially made on GRCh37 and transformed to GRCh38 to a callset made directly on GRCh38 may be due to either inherent callset differences or to errors in the transformation process. To understand the relative contributions of each, we took the GiaB benchmark callset on GRCh38, transformed it to GRCh37 using GenomeWarp, transformed that callset back to GRCh38 using GenomeWarp, and then compared the resulting callset to the original GiaB benchmark callset on GRCh38 using hap.py (**Supplementary Table S5**).

For both SNPs and indels, nearly all errors are false negatives (99.57% and 99.97%, respectively). This is expected owing to the conservative nature of the GenomeWarp algorithm; variants that cannot be confidently transformed are instead omitted along with the corresponding region. The presence of the 44 false positive variants was surprising given this design decision.

We investigated these cases in detail and in each case found that the error was due to the entire region surrounding a variant being mapped to a different location in the genome. For example, the region GRCh38.chr1:144515866-144515876 maps to GRCh37.chr1:144542180-144542190, but GRCh37.chr1:144542180-144542190 maps to GRCh38.chr1:149154796-149154807. When a variant is present in one of the GRCh38 regions but not the other a false variant is introduced. This error mode is due to the LiftOver chains not enforcing an injective mapping (visual example in **Supplementary Figure S2**) and is irreducible error for any tool relying on the accuracy of LiftOver chains as part of the transformation algorithm.

Comparison of GenomeWarp to mapping and calling variants directly

To investigate the difference in callsets generated by GenomeWarp versus mapping and calling on the target genome assembly, we used a dataset of Illumina HiSeq 2x250 bp reads of the HG003 sample generated for GiaB. The read data was aligned with Novoalign 3.02.07 (http://www.novocraft.com/products/novoalign/) to both the hs37d5 genome assembly and the GRCh38 genome assembly (available at ftp://ftp-trace.ncbi.nlm.nih.gov/giab/ftp/data/AshkenazimTrio/HG003_NA24149_father/NIST_Illumina_2x250bps/novoalign_bams/HG003.hs37d5.2x250.bam and ftp://ftp-trace.ncbi.nlm.nih.gov/giab/ftp/data/AshkenazimTrio/HG003_NA24149_father/NIST_Illumina_2x250bps/novoalign_bams/HG003.GRCh38.2x250.bam, respectively). DeepVariant 0.7.2 (Poplin et al, 2018) was used to call variants on both genome assemblies using its default parameters with the gVCF output option enabled.

The gVCF called directly on GRCh38 contained records spanning 2,837,235,359 bp of the genome. The gVCF called on hs37d5 was transformed to GRCh38 using GenomeWarp v1.2.0 with its default parameters, and the result spanned 2,798,332,413 bp, of which 2,792,373,870 bp were in common with the gVCF called directly on GRCh38 (a Jaccard similarity of 0.982126).

To understand the differences in callsets, four experiments were performed (**Supplementary Table S6**). First, the variants called on each of hs37d5 and GRCh38 were compared to the associated GiaB benchmarking callsets on those assemblies to verify the quality of the callsets prior to GenomeWarp transformation. SNP F1 scores exceeded 0.9994 and indel F1 scores exceeded 0.991 for both callsets, demonstrating the accuracy of DeepVariant. Next, the hs37d5 callset was transformed to GRCh38 using GenomeWarp. Evaluation of the resulting callset against the GiaB HG003 benchmarking callset on GRCh38 shows that the F1 scores decrease for both SNPs and indels (SNP F1 0.997323, indel F1 0.988256), owing mostly to losses in recall (**Supplementary Table S6**). The loss of recall can be attributed at least in part to the conservativeness of the GenomeWarp conversion algorithm as described in the above analysis on GenomeWarp transformation losses. The loss of precision is attributable to differences in the

callsets themselves, as the above analysis of GenomeWarp transformation losses shows that very few false positives are introduced by the transformation algorithm, and are consistent with the difference in total calls in the GiaB callsets on the two assemblies. Finally, the transformed callset was evaluated against the callset generated using DeepVariant directly on GRCh38 and filtering non-variant regions to those with a minimum GQ of 20. The resulting SNP F1 of 0.968662 and indel F1 of 0.972409 are substantially lower than the comparison to the GiaB benchmarking callset on GRCh38, showing that genomic regions outside the GiaB benchmark regions are enriched for differences between the assemblies that lead to both variant calling diversity and fewer successful liftover operations.

Reproducing the analyses of this manuscript

All data used in the analyses in this manuscript is publicly available at the links in **Supplementary Table S4** and above sections of the **Supplementary Note**. After building GenomeWarp v1.2.1 as described in https://github.com/verilylifesciences/genomewarp#building-this-project, it was run using the following command:

```
$ java -jar -Xmx20G verilylifesciences-genomewarp-1.2.1-runnable.jar \
  --lift_over_chain_path "$CHAIN" \
  --raw_query_vcf "$QUERY_VCF" \
  --raw_query_bed "$QUERY_BED" \
  --ref_query_fasta "$QUERY_FASTA" \
  --ref_target_fasta "$TARGET_FASTA" \
  --output_variants_file "$OUTPUT_VCF" \
  --output_regions_file "$OUTPUT_BED" \
  --work_dir "/tmp" \
  --intermediate_files
```

for the appropriate values of input variables.

After installing CrossMap v0.2.9 (http://crossmap.sourceforge.net/) as described at http://crossmap.sourceforge.net/#use-pip-to-install-crossmap, it was run using the following command:

```
$ python CrossMap.py vcf "$CHAIN" "$QUERY_VCF" "$TARGET_FASTA" "OUTPUT_VCF"
```

for the same values of input variables as used by GenomeWarp.

After downloading Picard v2.18.14 (https://github.com/broadinstitute/picard/releases/download/2.18.14/picard.jar), it was run using the following command:

```
java -jar picard.jar LiftoverVcf \
  I="$QUERY_VCF" \
  O="$TARGET_VCF" \
  CHAIN="$CHAIN" \
  R="$TARGET_FASTA" \
  REJECT="$REJECTED_VCF"
```

for the same values of input variables as used by GenomeWarp. The `RECOVER_SWAPPED_REF_ALT=true` flag was also applied in a separate instantiation of the tool.

Hap.py was run using the pre-built Docker image as described at https://github.com/Illumina/hap.py#docker. The command used was:

```
$ sudo docker run -it \
  -v "${DATA_DIR}:${DATA_DIR}" \
  -v "${OUTPUT_DIR}:${OUTPUT_DIR}" \
  pkrusche/hap.py /opt/hap.py/bin/hap.py \
  "$TRUTH_VCF" \
  "$TARGET_VCF" \
  -f "$TRUTH_BED" \
  -r "$TARGET_FASTA" \
  -o "$OUTROOT" \
  --engine=vcfeval
```

for the same values of $TARGET_VCF and $TARGET_FASTA as used by GenomeWarp, and the appropriate directory and truth set values.

Supplementary References

- Krusche, P et al. (2019) Best practices for benchmarking germline small variant calls in human genomes. *Nature Biotechnology*, in press. https://doi.org/10.1101/270157
- Poplin, R et al. (2018) A universal SNP and small-indel variant caller using deep neural networks. *Nature Biotechnology*, 36, 983-987.
- Zhao, H. et al. (2014) CrossMap: a versatile tool for coordinate conversion between genome assemblies. *Bioinformatics*, 30, 1006-1007.
- Zook, J.M. et al. (2014) Integrating human sequence data sets provides a resource of benchmark SNP and indel genotype calls. *Nature Biotechnology*, 32, 246-251.
- Zook, J.M. et al. (2016) Extensive sequencing of seven human genomes to characterize benchmark reference materials. *Scientific Data*, 3, 160025.
